# Long-Term Azithromycin Treatment in Pediatric Primary Ciliary Dyskinesia: A Retrospective Study

**DOI:** 10.3389/fped.2022.905253

**Published:** 2022-06-10

**Authors:** Yuhong Guan, Xiang Zhang, Haiming Yang, Hui Xu, Shunying Zhao

**Affiliations:** Department of Respiratory Medicine, National Clinical Research Center for Respiratory Diseases, Beijing Children’s Hospital, National Center for Children’s Health, Capital Medical University, Beijing, China

**Keywords:** primary ciliary dyskinesia, pediatric, azithromycin, treatment, lung function

## Abstract

**Objectives:**

Primary ciliary dyskinesia (PCD) is a rare genetic disease mainly involved in lung dysfunction. PCD patient outcomes after azithromycin (AZM) treatment have rarely been reported. This study was aimed to assess AZM treatment effects on disease progression of pediatric PCD patients.

**Study Design:**

This retrospective follow-up study involved PCD patients diagnosed from 2009 to 2021. Changes of clinical outcomes, pulmonary function, and chest computed tomography findings were compared between untreated and AZM-treated patients.

**Results:**

Of 71 enrolled patients (median follow-up duration of 3.1 years), 34 received AZM (AZM-treated group) and 37 received no AZM (AZM-untreated group). At diagnosis, no significant intergroup differences in age, sex, height, weight, number of respiratory exacerbations, and FEV1% and FVC% predicted values were found, although FEF_25–75_% predicted was lower in AZM-treated group. Between treatment initiation and follow-up, patients in AZM-treated group had less respiratory exacerbations than that of AZM-untreated group (mean ± SD, 1.4 ± 0.8 vs. 3.0 ± 2.1, times/year *P* = 0.001) and fewer AZM-treated group patients exhibited exercise intolerance. Increases above baseline of AZM-treated FEV1% and FVC% predicted values exceeded that of AZM-untreated group, but intergroup differences were insignificant (FEV1% predicted: (median, IQR) 5.3 [−13.4, 9.4] vs. 1.8 [−12.1, 9.5], *P* = 0.477; FVC% predicted: (median, IQR) 6.7 [−7.6, 18.8] vs. 1.6 [−5.6, 7.6], *P* = 0.328).

**Conclusion:**

Long-term AZM treatment can reduce respiratory infection frequency and may maintain pulmonary diseases stable in pediatric PCD patients with worse lung function.

## Introduction

Primary ciliary dyskinesia (PCD), a rare genetic disorder that is characterized by recurrent respiratory infection and pulmonary dysfunction, has historically been viewed as a mild respiratory condition (1). However, recent data suggest that PCD disease can seriously impair lung function in children of preschool age. In fact, in some children PCD can worsen rapidly and lead to greater decline in lung function during childhood than occurs in pediatric cystic fibrosis (CF) patients (2–4). Frequent respiratory tract infections appear to be important factor of pulmonary disease progression in PCD patients (5), while also causing repeated hospitalizations that adversely affect quality of life of patients. Moreover, results of one European study revealed that 37% of PCD patients who did not receive regular treatment accumulated more than 40 outpatient visits before diagnosed (6).

Respiratory management of PCD patients is critical in order to prevent irreversible lung damage and appropriate treatment will likely prevent or slow progression of lung damage once a diagnosis is established (5). Current PCD treatment mainly follows CF and non-CF bronchiectasis treatment recommendations, with azithromycin (AZM) maintenance therapy known to exert beneficial anti-inflammatory effects when use to treat CF and non-CF bronchiectasis (7, 8). RCTs examining azithromycin versus placebo demonstrated increases of percent predicted forced expiratory volumes (ranged from 2.95 to 6.2%) in AZM-treated CF patients and two studies showed similar results of azithromycin treatment in adult non-CF bronchiectasis lasting 6 and 12 months (9, 10). For a long time, there were only anecdotal reports of benefit from macrolide antibiotic (Clarithromycin, erythromycin, and azithromycin) in PCD patients (11, 12). Recently, an international BEAT-PCD consensus statement for infection prevention and control was published in order to improve diagnosis and treatment (13). Besides, the recently well-designed randomized, placebo-controlled trial showed azithromycin maintenance therapy for 6 months was well tolerated and halved the rate of respiratory exacerbations, while measurements of pulmonary function indicators revealed no significant intergroup differences in changes of percent of predicted FEV1, FVC, and FEF_25–75_ (14). Nonetheless, treatment outcomes of pediatric PCD patients receiving AZM are unclear, since studies abovementioned conducted to date have been focused either on adults or included both children and adults.

Here, we describe clinical outcomes of AZM-treated pediatric PCD patients based on longitudinal follow-up periods of 3.1 years. The aim of the study was to evaluate effects of long term AZM treatment on pulmonary disease status of pediatric PCD patients through retrospective analysis of follow-up clinical data conducted at the largest pediatric pulmonary treatment center in China.

## Subjects and Methods

### Subjects

This study was conducted in order to provide a retrospective analysis of clinical data obtained from a group of PCD pediatric patients who were diagnosed in the Department of Respiratory Medicine in Beijing Children’s Hospital between January 2009 and July 2021. PCD patients enrolled in this study had received definitive PCD diagnoses and consented to receive follow-up care. The study was approved by the Beijing Children’s Hospital Ethics Committee. Verbal and written consent was obtained from all parents and pediatric patients (≥8 years).

### Primary Ciliary Dyskinesia Diagnosis

Primary ciliary dyskinesia diagnostics remains challenging. PCD diagnosis in this study was based on at least one of the following criteria: patients with recognized ciliary ultrastructural defect or biallelic pathogenic variants in a PCD-associated gene; or at least two of the four key clinical features (namely, unexplained neonatal respiratory distress in a term infant; year-round daily cough or nasal congestion beginning before 6 months of age; organ laterality defect) combined with low nasal nitric oxide (nNO) level (excluding CF). Kartagener syndrome, which was considered to be PCD even if the previously mentioned criteria were not fulfilled completely (15, 16). Detailed methods of TEM, next generation sequencing and nasal NO were as described in previous study (16).

### Data Collection

Baseline clinical data were collected through electronic medical records that included demographic information: name, sex, date of birth, age at diagnosis, height at diagnosis, weight at diagnosis, medical history, diagnostic information, lung function, and chest imaging results, history of hospitalizations and drug treatments, antibiotics used to treat acute infections (dosage and treatment duration), prophylactic antibiotics (antibiotic drugs, dosage, and start date), other prescribed medications (e.g., mucolytic agents, corticosteroids), and surgeries. Follow-up data were collected using a pre-designed form that included the annual frequency of respiratory infection-associated exacerbations, lung function test results, chest computed tomography (CT) imaging findings, growth measurements, days off from school due to PCD, sports endurance, etc.

Clinical data of patients were compared according to AZM treatment duration. Patients treated regularly with AZM at a dose of 10 mg/kg administered 3 times per week for longer than 3 months were assigned to the AZM-treated group, while those who did not take AZM or less than 3 months were assigned to AZM-untreated group. Clinical changes, frequency of respiratory exacerbations, changes in height and weight, lung function changes, and changes in chest imaging findings were compared between the two groups.

### Statistics

Statistical analyses were performed using the Statistical Package for the Social Sciences (SPSS) version 20.0 (SPSS Inc, Chicago, IL, United States). Continuous data were expressed as the mean ± standard deviation or median (interquartile range, IQR). The normality of each variable distribution was assessed using the Kolmogorov-Smirnov test. Comparisons of quantitative data between AZM-treated and AZM-untreated groups were conducted using the *T*-test or Mann-Whitney *U* test, while *X*^2^ or Fisher’s exact tests were used to compare proportions. Statistical tests were two−sided and were deemed statistically significant for *P* < 0.05.

## Results

### Demographics and Clinical Characteristics of Patients at Baseline

From January 2009 to July 2021, a total of 83 patients with confirmed PCD diagnoses were admitted to the Department of Respiratory Medicine, Beijing Children’s Hospital. Clinical and genetic characteristics of patients were collected and analyzed during the follow-up period. Of the total of 83 PCD patients, 71 patients received follow-up care, seven patients were lost to follow-up, four refused to participate, and one patient died of severe complex congenital heart disease at 1 year of age. In summary, follow-up data were obtained from a total of 71 patients, of which 39 patients were males and 32 were females; the overall median duration of the follow-up period after diagnosis was 3.1 years (ranging from 4 months to 9.4 years) and the average age at diagnosis was 7.6 ± 3.3 years.

Age, sex, and clinical characteristics between AZM-treated and AZM-untreated groups of PCD patients were very similar at baseline [Table 1, partly reported in previous studies (16)] except for FEF_25–75_% predicted, which were lower in the AZM-treated group than in the AZM-untreated group (mean, SD, 46.9 ± 20.8 vs. 70.8 ± 28.0, *P* = 0.008, respectively).

**TABLE 1:** Baseline characteristics of patients by treatment group.

	AZM-treated (*n* = 34)	AZM-untreated (*n* = 37)	*P*-value
Gender (male)	20 (59%)	23 (62%)	0.812
Age (mean, SD, years)	7.9 (3.4)	8.1 (3.1)	0.844
Height (mean, SD, percentile)	45.9 (27.9)	55.7 (28.6)	0.200
Weight (mean, SD, percentile)	33.1 (31.0)	36.8 (26.9)	0.599
BMI (mean, SD, percentile)	20.7 (28.6)	24.2 (26.3)	0.636
BMI (mean, SD, Zscore)	-1.67 (1.82)	-1.14 (1.18)	0.208
**Pulmonary function**			
FEV1% predicted (mean, SD)	75.4 (24.4)	87.4 (20.3)	0.118
FVC% predicted (mean, SD)	81.5 (21.4)	89.6 (17.1)	0.217
FEF_25–75_% predicted (mean, SD)	46.9 (20.8)	70.8 (28.0)	0.008
**Bacteria in sputum cultures**			0.940
Streptococcus pneumoniae (*n*)	12	12	
Haemophilus influenzae (*n*)	10	8	
Pseudomonas aeruginosa (*n*)	3	3	
Staphylococcus aureus (*n*)	2	3	
Number lobes of bronchiectasis (mean, SD)	2.14 (1.04)	2.8 (1.03)	0.838
nNO(mean, SD, nl/min)	39.9 (45.5)	40.1 (42)	0.754
Genotype	*DNAH11* (5);*CCDC39* (4);*DNAH5* (4);*DNAAF3,DNAH1*, *RSPH4A, HEATR2*, *RSPH9*, (respectively 2);*CCDC40*, DNAAF2, *CCNO* (respectively one),negative (6),not test (2)	*DNAH11* (7);*DNAH5* (4);*DNAH9*, *CCDC40 (2)*, *CCNO*, *DNAI1*, *LRRC6*, *RSPH4A*, *SPAG1*, *DNAAF3*, *DNAH1* (respectively 1);negative (6),not test (9)	

*BMI, body mass index; FEV1, forced expiratory volume in one second; FVC, forced vital capacity; FEF_25–75_, forced expiratory flow at 25–75%.*

### Primary Ciliary Dyskinesia Management

In this study, routine therapies used to treat PCD patients included long-term AZM administration, airway clearance (daily chest physiotherapy), inhaled hyperosmolar agents, and corticosteroids. Treatment during acute infection included antibiotics, with AZM administered most often, followed by ceftriaxone, ceftazidime, cefoperazone sodium, sulbactam sodium, and meropenem. PCD patients presenting with wheezing as the major symptom were also treated with intravenous or oral corticosteroids.

Treatment management details of 71 patients are shown in Table 2. Although long-term oral AZM was recommended for 90% of patients with definite PCD diagnosis, only about 50% of patients were able to adhere to long-term oral AZM administration, of which 46% complied with recommended airway clearance physiotherapy.

**TABLE 2:** Management of 71 children with PCD.

Parameter	AZM-treated (*n* = 34)		AZM-untreated (*n* = 37)	*P*-Value
		
Azithromycin		Follow-up duration (mean, SD, year)		
0–3 months	0		37 (100%, 37/37)	
3–6 months	9 (26.5%, 9/34)	1.9 (0.7)	0	
6–12 months	12 (35.3%, 12/34)	2.6 (0.6)	0	
≥12 months	13 (38.2%, 13/34)	3.1 (1.0)	0	
Adverse event (Gastrointestinal discomfort)	3 (9%, 3/34)		2 (5%, 2/37)	0.340
Mucolytic agents	35.3% (12/34)		32.4% (12/37)	0.799
Eucalyptus pinene capsule, *n*	9		6	
*N* -acetylcysteine, *n*	2		5	
Ambroxol hydrochloride, *n*	1		1	
Nebulized hypertonic saline	4		1	0.136
Sino-nasal rinse (saline)	3 (9%, 3/34)		3 (8%, 3/37)	0.914
Inhaled corticosteroids	12% (4/34)		11% (4/37)	0.899
Budesonide/formoterol, *n*	3		3	
Fluticasone/salmeterol, *n*	1		1	
bronchoalveolar lavage	5 (15%, 5/34)		3 (8%, 3/37)	0.380
Surgery	4 (13.3%)		3 (9%)	0.606
Pulmonary lobectomy	2		1	
pectus excavatum correction	0		1	
Surgery for congenital heart disease	2		1	

Ultimately, the average duration of oral AZM treatment was 14 months (range 0.5–29 months). Notably, in the total of follow-up patients, 34 (48%) of patients continually took AZM for longer than 3 months, including nine patients who took AZM for 3–6 months and 25 patients for more than 6 months. The remaining 37 (52%) patients who either did not regularly take AZM or took AZM for less than 3 months were assigned in the AZM-untreated group, including 26 patients who did not take AZM, four patients who regularly took AZM for 2 weeks, four patients who took AZM for 1 month, and two patients who took AZM for 2 months. Aside from AZM treatment, there were no significant differences in other treatment measures between the two groups (Table 2).

In the AZM-treated group, three patients experienced transient nausea and abdominal pain that ceased after AZM administration was changed to oral after meals. No other serious adverse reactions were observed. Moreover, testing at intervals during follow-up revealed that no patients suffered from leucopenia or neutropenia, and no patients had abnormal alanine transaminase levels, or abnormal electrocardiogram findings.

### Clinical Outcomes

#### Growth Changes

Average percentage-based increases in height of children were 9.6 and 6.9% for AZM-treated and AZM-untreated groups, respectively. From the time of initial diagnosis to the final follow-up visit, height of 75% of patients were increased in AZM-treated group as compared to 64% of patients in AZM-untreated group. Meanwhile, BMI percentile increases did not significantly differ between AZM-treated and AZM-untreated groups [median, IQR (1.5, 1.0–2.0) vs. (1.7, −6.4 to 17.9), respectively], while the average weight in AZM-treated group increased by only 5.1% as compared to 10.2% for the AZM-untreated group (Table 3), which were also of no significance of difference.

**TABLE 3:** Clinical outcomes at follow-up of patients by treatment group.

Parameter	AZM-treated (*n* = 34)	AZM-untreated (*n* = 37)	*P*-value
Age (years) (mean, SD)	10.8 (3.6)	11.5 (3.5)	0.412
Follow up duration (mean, SD, year)	2.7 (1.7)	3.5 (2.1)	0.097
Changes of height (percentile) (median, 25th; 75th centile)	9.6 (−0.2, 22.0)	6.9 (−17.8, 24.5)	0.344
Increased (*n*, %)	18 (75)	14 (64)	0.403
Decreased (*n*, %)	6 (25)	8 (36)	
Changes of weight (percentile) (median, 25th; 75th centile)	5.1 (−0.0, 22.0)	10.2 (−3.0, 28.2)	0.800
Changes of BMI (percentile) (median, 25th; 75th centile)	1.5 (1.0, 2.0)	1.7 (−6.4, 19.7)	0.085
Changes of BMI (Z score) (mean, SD)	0.44 (1.0)	0.67 (1.4)	0.522
**Changes of pulmonary function**			
FVC% predicted (median, 25th; 75th centile)	6.7 (−7.6, 18.8)	1.6 (−5.6, 7.6)	0.328
FEV1% predicted (median, 25th; 75th centile)	5.3 (−13.4, 9.4)	1.8 (−12.1, 9.5)	0.477
FEF_25–75_% predicted (median, 25th; 75th centile)	6.0 (−18.4, 16.4)	−2.8 (−20.3, 11.2)	0.594
Increased (%)	75%	60%	0.638
Decreased (%)	25%	40%	
**Bronchiectasis on HRCT**			0.180
Aggravated or newly (*n*, %)	1 (5)	4 (25)	
Improved (*n*, %)	7 (35)	3 (19)	
Stable (*n*, %)	12 (60)	9 (56)	
Respiratory exacerbations (times/year)	1.4(0.8)	3.0 (2.1)	0.001
Reduction of sputum volume	93% (26/28)	70% (21/30)	0.043
Exercise intolerance*	12% (3/26)	24% (7/29)	0.009

HRCT, High resolution computed tomography. *That is delineating physical activity patterns described by parents or child caretakers including shorter time of daily free play or short of breath after strenuous activities such as climbing 6 or more floors of stairs or frequent rest during running games compare to heathy children. This had been clarified in the method section.

#### Primary Ciliary Dyskinesia Masnifestations

The mean frequency of respiratory exacerbations was significantly reduced in the AZM-treated group as compared to the AZM-untreated group (mean, SD, 1.4 ± 0.8 vs. 3.0 ± 2.1, respectively, *P* = 0.001). In addition, significantly more patients got decreased sputum volumes in AZM-treated group as compared to that of AZM-untreated group (93% vs. 70%, respectively, *P* = 0.043). Moreover, number of percentages of AZM-treated group patients with exercise intolerance was lower than that of the AZM-untreated group (12% vs. 24%, respectively, *P* = 0.009).

#### Changes in Chest Computed Tomography Findings and Pulmonary Function Tests Results

Only 36 patients (20 in AZM-treated group and 16 in the AZM-untreated group, respectively) received repeated chest CT scans. Scans of one patient in AZM-treated group revealing aggravation of lung damage with pathological involvement of new bronchiectasis, while, results obtained for seven patients showed signs of lung improvement and results for 12 patients showed no obvious change in CT findings, number of which in the AZM-untreated group were 4, 3, and 9 (Table 3), respectively.

Results of cross-sectional analysis of lung function-based measurements collected during the follow-up period are shown in Table 3. The first measured FEV1% predicted of patients at baseline varied widely between normal and subnormal values (i.e., <80% of predicted), with a substantial number (*n* = 13; 33%) demonstrating subnormal values (59–79% of predicted). Importantly, lung function indicator values seemed increased after AZM treatment, but of no significance of difference, with increases by 5.3% and 1.8%, respectively in AZM-treated and AZM-untreated groups, respectively. Meanwhile, although the FEF_25–75_% predicted value in the AZM-treated group was significantly lower than that of the AZM-untreated group at baseline, intergroup differences of changes of FEF_25–75_% predicted were of no statistically significance [median, IQR, 6.0 (−18.4 to 16.4) vs. −2.8 (−20.3, 11.2), respectively, *P* = 0.594].

### Microbiology Data

Numbers of positive sputum culture results varied among patients. *Streptococcus pneumoniae* was the most commonly isolated pathogen (65% of sputum specimens resulted in at least one isolation), followed by *Haemophilus influenzae* and *Staphylococcus aureus* (Table 3). *Pseudomonas aeruginosa* was isolated at least once from sputum only obtained from five different subjects (two cases in AZM-treated group and three in AZM-untreated group). Among patients with positive sputum culture results, sputum of only eight patients were retested, including one patient whose sputum yielded a new *P. aeruginosa* isolate and two patients whose sputum tested negative for *S. pneumoniae*. Due to the small number of patients with repeated sputum culture test results, the differences between AZM-treated and AZM-untreated groups were insignificant.

## Discussion

Primary ciliary dyskinesia is a lifelong disease that can promote occurrence of recurrent airway infections that finally result in bronchiectasis and declining lung function that, in turn, support development of respiratory infections and perpetuate a vicious cycle of disease. Interrupting this vicious cycle is vital in order to delay disease progression. In fact, results of several studies have shown that AZM treatment of patients with CF and non-CF bronchiectasis for 3–6 months improved lung function and nutritional status, while also reducing C-reactive protein levels, pulmonary exacerbations, and hospitalization rates (7–10), but only one multicenter phase 3 trial study was performed that showed similar results included both pediatric and adult PCD patients (14). Here, we retrospectively studied a school-age pediatric population with a mean age at baseline of 8.0 years and a mean age at follow-up of 11.03 years, parts of baseline data had been reported in previous study (16). Our results revealed that pediatric PCD patients with long-term AZM use experienced significantly fewer respiratory excerbations than those who had not taken AZM, as observed in the previous study (14).

Azithromycin is currently widely used in chronic respiratory diseases including PCD (17), due to its ability to alleviate both infection and inflammation. More specifically, AZM plays an anti-infective role by inhibiting synthesis of bacterial proteins that can interfere with bacterial biofilm generation and production of other virulence factors (18). Concurrently, AZM plays an anti-inflammatory role by inhibiting host production of IL-8 and tumor necrosis factor-α (TNF-α) (19, 20). Notably, the decreased frequency of respiratory excerbations in the AZM-treated group as compared to the control group may be related to relatively reduced daily sputum volume associated with AZM treatment effects on mucus properties and mucus production (21). In addition, our results also revealed that PCD patients who received long-term AZM therapy exhibited less chance of exercise intolerance as compared to control group. It is showed that a history of long-term AZM administered three times per week reduced the frequencies of inpatient and/or outpatient doctor visits for respiratory infections, while also improved school attendance and overall quality of life.

Studies have shown that early in life, impairment of lung function of PCD patient begins and increases with age, with magnitudes of PCD disease-induced effects on FEV1 and FVC values eventually resembling those reported in studies of CF patients (22, 23). However, a few of studies have investigated macrolide antibiotic treatment effects on FEV1 values of non-CF bronchiectasis produced conflicting results (24–26), with only one of the studies indicating improvement of FEV1 after treatment (24). In this study, lung function varied widely among our patients and the mean follow-up time was 3.1 years and longest follow-up duration was 9.4 years. Importantly, baseline FEF_25–75_% predicted of patients in the AZM-treated group were lower than that in corresponding control group. Moreover, AZM-treated group lung function improved slightly more than that of the AZM-untreated group, although the intergroup difference was of no apparent difference, due to small numbers of patients had lung function test results available both at baseline and follow-up. Ultimately, the results collectively suggest that regularly long term AZM use may stabilized FEV1, FVC, and FEF_25–75_ in patients with poor lung function or more severe disease resulting from frequent respiratory infections.

In fact, that lung spirometry is less sensitive than high-resolution computed tomography (HRCT) at detecting functional and structural lung damage induced by PCD disease (27). Thus, CT scans were used here to assess lung damage in our PCD patient cohort. Results of this study, which were of no statistically significant differences at baseline between the two groups, revealed that about 90% of patients in the AZM-treated group exhibited signs of stable or improved lung function as compared to about 75% of that in control group patients. However, this change did not differ apparently between the two groups which need to be confirmed by more detailed and rigorously designed studies due to the evaluation complexity of imaging changes.

Importantly, in a large group of over 3,000 PCD patients, Goutaki et al. found that both growth and nutrition are affected adversely from early life in PCD patients with delayed diagnosis (28). However, other studies showed preschool referral to a PCD center was not associated with better BMI (29). Our results revealed that BMI was a little lower in both groups at baseline which may be associated with late diagnosis in this cohort, while the mean heights were in normal range. Notably, although the average follow-up duration of the AZM-treated group was shorter than that of the control group, the percent increase in height of the AZM-treated group was slightly greater than that of controls, but the intergroup difference was of no statistical significance. Indeed, at first measurement, most PCD patients in this study were of normal height and weight. Thus, these indicators would not likely increase further with AZM treatment during follow-up.

The most common adverse reactions associated with AZM treatment which were observed in this study were that occasional and mild gastrointestinal reactions experienced only in three patients who received regular AZM treatment. Importantly, such reactions improved after patients adjusting the timing of AZM administration. Although hearing decrements were observed to be more common in the azithromycin group in a 12-month RCT in patients with chronic obstructive pulmonary disease (30), no serious adverse reactions, such as ototoxicity and cardiotoxicity, were observed.

A limitation of this retrospective study that lung function tests, chest CT scans, and sputum cultures were not regularly conducted during follow-up duration, which indicate that inadequate patient education and inconsistencies of disease management were exist in China. Indeed, all patients with bronchiectasis or chronic wet cough were meant to take Azithromycin. Overall, only about 50% patients adhere to recommended treatment. There may be several reasons. First, the lack of professional physiotherapists in China has led to assignment of PCD treatment management responsibilities to respiratory doctors, nurses, and parents. Second, most parents of pediatric PCD patients didn’t recognize the importance of long-term treatment of all sorts. Besides, parts of them were worried about side effects to their children. All these factors contribute to poor adherence to recommended airway clearance measures, drugs, and other treatment sorts. In the future, establishment of PCD follow-up treatment programs at medical research centers with standardized prospective collection of data related to characteristics and factors associated with PCD phenotypes may help to provide more scientifically based and comprehensive disease management evidence.

## Data Availability Statement

The original contributions presented in this study are included in the article/supplementary material, further inquiries can be directed to the corresponding author.

## Ethics Statement

The studies involving human participants were reviewed and approved by Beijing Children’s Hospital Ethics Committee. Written informed consent to participate in this study was provided by the participants’ legal guardian/next of kin.

## Author Contributions

YG and SZ contributed to conception and design of the study and organized the database. YG, XZ, HX, and HY collected the data. YG performed the statistical analysis and wrote the first draft of the manuscript. All authors read and approved the final manuscript.

## Conflict of Interest

The authors declare that the research was conducted in the absence of any commercial or financial relationships that could be construed as a potential conflict of interest.

## Publisher’s Note

All claims expressed in this article are solely those of the authors and do not necessarily represent those of their affiliated organizations, or those of the publisher, the editors and the reviewers. Any product that may be evaluated in this article, or claim that may be made by its manufacturer, is not guaranteed or endorsed by the publisher.

## Abbreviations

AZM: azithromycin
BEAT-PCD: better experimental approaches to treat PCD
CF: cystic fibrosis
FEF_25–75_: forced expiratory flow at 25–75% of FVC
FEV1: forced expiratory volume in one second
FVC: forced vital capacity
PCD: primary ciliary dyskinesia.
